# Non-quaternary oximes detoxify nerve agents and reactivate nerve agent-inhibited human butyrylcholinesterase

**DOI:** 10.1038/s42003-021-02061-w

**Published:** 2021-05-14

**Authors:** Gabriel Amitai, Alexander Plotnikov, Shira Chapman, Shlomi Lazar, Rellie Gez, Dan Loewenthal, Khriesto A. Shurrush, Galit Cohen, Leonardo J. Solmesky, Haim Barr, Alan J. Russell

**Affiliations:** 1 The Wohl Drug Discovery Institute, Grand Israel National Center for Personalized Medicine (G-INCPM), The Weizmann Institute of Science, Rehovot, Israel; 2 Department of Pharmacology, Israel Institute for Biological Research (IIBR), Ness Ziona, Israel; 3 Department of Analytical Chemistry, IIBR, Ness Ziona, Israel; 4 Department of Chemical Engineering, Carnegie Mellon University, Pittsburgh, PA USA

**Keywords:** Chemical libraries, Biologics

## Abstract

Government-sanctioned use of nerve agents (NA) has escalated dramatically in recent years. Oxime reactivators of organophosphate (OP)-inhibited acetylcholinesterase (AChE) and butyrylcholinesterase (BChE) serve as antidotes toward poisoning by OPNAs. The oximes used as therapeutics are quaternary compounds that cannot penetrate the blood–brain barrier (BBB). There remains an urgent need for the development of next generation OPNA therapeutics. We have developed two high-throughput screening (HTS) assays using a fluorogenic NA surrogate, *O*-ethyl methylphosphonyl *O*-4-methyl-3-cyano-coumarin (EMP-MeCyC). EMP-MeCyC detoxification and EMP-BChE reactivation screening campaigns of ~155,000 small molecules resulted in the identification of 33 nucleophile candidates, including non-quaternary oximes. Four of the oximes were reactivators of both Sarin- and VX-inhibited BChE and directly detoxified Sarin. One oxime also detoxified VX. The novel reactivators included a non-quaternary pyridine amidoxime, benzamidoxime, benzaldoxime and a piperidyl-ketoxime. The VX-inhibited BChE reactivation reaction rates by these novel molecules were similar to those observed with known bis-quaternary reactivators and faster than mono-quaternary pyridinium oximes. Notably, we discovered the first ketoxime reactivator of OP-ChEs and detoxifier of OPNAs. Preliminary toxicological studies demonstrated that the newly discovered non-quaternary oximes were relatively non-toxic in mice. The discovery of unique non-quaternary oximes opens the door to the design of novel therapeutics and decontamination agents following OPNA exposure.

## Abstract (web summary)

Amitai et al. identified 4 potential small molecule antidotes toward nerve agents poisoning by high-throughput-screening of over 150,000 compounds. Unique non-quaternary oximes represent new potential antidotes for future development as they detoxified nerve agents and also reactivated nerve agent-inhibited butyrylcholinesterase.

## Introduction

The use of lethal nerve agents in targeted and indiscriminate attacks by government and non-government actors has heightened the need for effective therapies and decontaminating systems. Organophosphorus (OP) insecticides or chemical warfare nerve agents (NA) cause rapid and covalent inhibition of acetylcholinesterase (AChE) and accumulation of excessive levels of acetylcholine (ACh) in cholinergic synapses. If left untreated, acute OP poisoning causes dose-dependent toxicity and eventually rapid death^1^. The current treatment of OP poisoning combines the use of oxime reactivators of OP-AChE, anticholinergic drugs such as atropine and anti-convulsants such as diazepam or midazolam^2^. The discovery of 2-PAM as a reactivator of OP-AChE^3^ led to numerous mono- and bis-quaternary oximes AChE reactivators with activity in vitro and in vivo. 2-PAM, obidoxime, and HI-6 (Supplementary Scheme 1 and Supplementary Notes) were fielded as OP nerve agents (OPNAs) antidotes for combat soldiers around the world^4^. More recently, quaternary imidazolium oximes^5^ were developed as reactivators of OP-inhibited AChE and its enzyme congener butyrylcholinesterase (BChE). Compared to tertiary nitrogen analogs, positively charged quaternary nitrogens increase the affinity of oximes to the anionic sub-site of AChE and BChE, and decrease the pK_a_. At physiological conditions, reduced oxime pK_a_ leads to higher levels of reactive oximate anion^6^. However, quaternary oximes are not able to cross the blood–brain barrier (BBB), thus limiting their therapeutic efficacy. BBB-permeable oximes with lipophilic quaternary oximes^7^ or oximes bearing a tertiary nitrogen^8,9^ are therefore of great interest as efficacious antidote designs.

Following a successful reactivation of AChE and BChE, the enzyme may be further challenged by blood-circulating free OPs and phosphoryl-oxime intermediates. To counteract this toxic rebound effect, we have previously developed small molecule bifunctional hybrid scavengers that contain both oxime and hydroxamate nucleophiles in the same molecule^10^. Similarly, countermeasures for toxic OPs in the blood can be developed from bioscavengers such as exogenous AChE and BChE^11^. Enzyme-based universal bioscavengers are broadly effective for a wide variety of OPNAs such as Novichok NAs used in assassinations attempts^12,13^. As a clinical stoichiometric scavenger, 200–500 mg BChE is required for protection against ~1xLD_50_ of V and G OPNAs in humans^14^. In mice, BChE with a co-applied oxime reactivator provided catalytic bioscavenging toward a variety of OPNAs, requiring significantly lower doses of BChE (0.5–1 mg/kg)^6,12^. However, an effective emergency care therapeutic requires the small molecule and protein components to maintain a similar duration of activity in vivo and overcome the significant disparity between the pharmacokinetics (PK) of free oxime and BChE in blood. In order to bridge this PK gap, we are interested in the direct conjugation of oximes to polymers. Such protein polymer–oxime conjugates were recently synthesized in our lab^15^. These polymer–oxime adducts tethered covalently to BChE by the atom transfer radical polymerization (ATRP) process were shown to reactivate OP-inhibited BChE by inter- and intramolecular interactions^15^.

In order to expand the chemical space of OPNA therapeutics and decontaminants, we have now employed high-throughput screening (HTS) to drive the discovery of new OP detoxifiers and OP-BChE reactivators. Since certain nucleophilic compounds could react directly with toxic OPs and thereby degrade them, they are defined as OP detoxifiers. The nucleophiles that could displace the covalently bound organophophoryl moiety from the active site serine residue of BChE, are defined as OP-BChE reactivators. Accordingly, two HTS assays for detoxification and reactivation were deployed in screens of ~150,000 small molecules leading to 19 detoxification and 14 reactivation compounds, respectively. Further validation studies confirmed detoxification of the NAs VX and Sarin, as well as reactivation of VX- and Sarin-inhibited BChE by four non-quaternary oximes of unique structures. Preliminary acute toxicity studies in mice indicate the compounds are relatively non-toxic and safe for further in vivo studies.

## Results and discussion

### Detoxification screen

In an initial screen of 152,304 compounds, we used both commercial screening libraries and a unique nucleophile-focused compound library selected from Enamine and academic collections (see “Methods” section). The nucleophile library was selected by a chemoinformatics search of available compounds containing NOH and resulted in a set enriched in aldoximes, ketoximes, amidoximes, hydroxamates, alkyl- and amino-hydroxamates. A robust primary assay (*Z*′ = 0.7) was established using a fluorogenic surrogate of OPNA for detoxification: *O*-ethyl methylphosphonyl 4-methyl-3-cyano-coumarin (EMP-MeCyC). Interference compounds (fluorescent enhancers or quenchers) were eliminated by monitoring instantaneous changes in the fluorescent emission of MeCyC-OH in presence of compounds. The nucleophile-focused library was highly enriched in confirmed EMP-MeCyC detoxifiers (>40% of BHA-induced fluorescence^10^) as compared to the commercial collection. In addition to the above filtering criteria, known nucleophiles and quaternary compounds were discarded, leaving 19 compounds for further investigation (Supplementary Fig. 1a). Further validation was performed with nerve agents Sarin and VX by measuring detoxification of Sarin, and reactivation of Sarin-BChE and VX-BChE conjugates. The top 4 compounds displayed both activities as Sarin detoxifiers and Sarin/VX-BChE reactivators (Supplementary Fig. 1b).

### Reactivation screen

A parallel screening approach was used to identify new reactivators of EMP-MeCyC-inhibited BChE (Supplementary Fig. 2a), using Resorufin Pivaloate (RP) as a fluorogenic substrate. The same chemical libraries as in the detoxification screening were tested as reactivators. Briefly, an inhibited BChE-EMP-MeCyC covalent complex was formed prior to dilution with library compounds, followed by BChE activity assays with RP as the substrate. The phenomenon of direct oximolysis of substrates such as RP is known and can contribute to high background fluorescence. Therefore, following the primary screen, candidate reactivators were tested once more with a four-fold dilution of regenerated BChE prior to performing the enzyme activity step. In this way, the most potent reactivators were selected with minimal oximolysis of RP (see “Methods” section and Supplementary Fig. 2a). As in the detoxification screen, the nucleophile-focused library was highly enriched for hit compounds and demonstrated the utility of a curated library coupled with high-throughput screening. For orthogonal confirmation of reactivators, an independent absorbance-based assay was developed. The Ellman method quantified the chromophoric formation of BChE hydrolysis product thiocholine-DTNB (see “Methods” section and Supplementary Fig. 2a). The nucleophiles (14 in total) that were discovered in the screen were confirmed as reactivators and were promoted to OPNA-inhibited BChE studies. Four compounds displayed both Sarin detoxification and Sarin/VX-BChE reactivation potency (Supplementary Fig. 2b).

### Validation of nucleophiles for reactions with VX and Sarin

We were gratified by the results of the two HTS campaigns targeting the discovery of novel detoxification and reactivation molecules. Each campaign was performed with more than 150,000 small molecules, which, to our knowledge, is the largest such screen for OPNA countermeasures to date. From over half a million individual experiments, the HTS campaigns resulted in the identification of 19 oximes for the detoxification of EMP-MeCyC a fluorogenic VX surrogate, as well as 14 oxime reactivators of EMP-MeCyC-inhibited-human BChE. Next, we tested each of the hit compounds for their kinetic potency as VX- and Sarin-detoxifiers, as well as VX- and Sarin-inhibited-BChE reactivators.

**Fig. 1:**
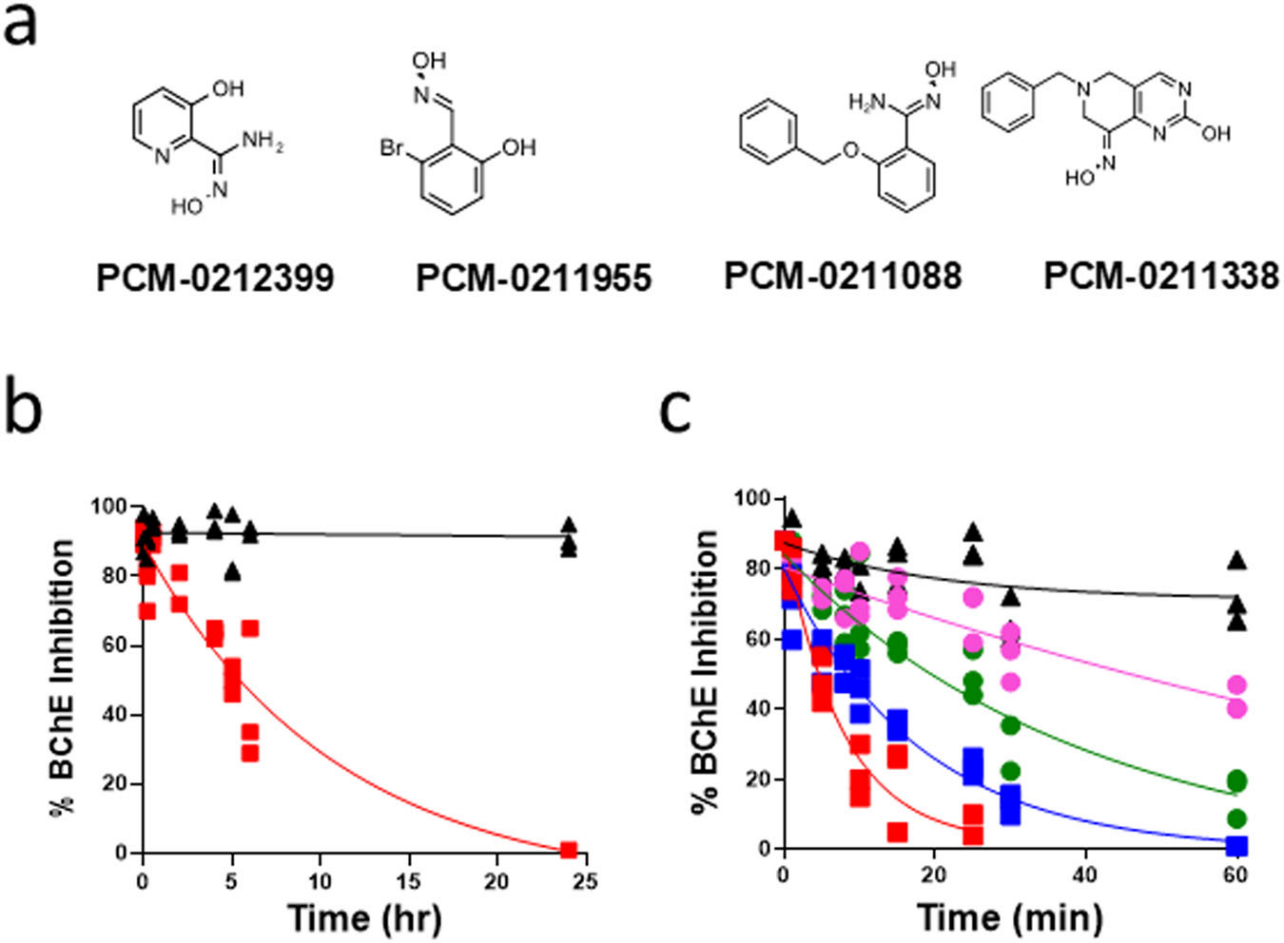
Structure of HTS-discovered oximes and nerve agent detoxification by oximes. **a** Chemical structure of HTS-discovered oximes (IUPAC names appear in Supplementary Information page 1 under Supplementary Scheme 1). **b** Time-Course of VX detoxification by PCM-0212399, VX detoxification measured by enzyme rescue assay. [VX] = 1 µM [PCM-0212399] = 0.5 mM (red squares). **c** Sarin detoxification by PCM-0212399 (red squares), PCM-0211955 (blue squares) PCM-0211338 (green circles), and PCM-0211088 (purple circles) measured by the enzyme rescue assay. [Sarin] = 1 µM, [Oximes] = 0.5 mM, 20 mM phosphate pH = 8.0 (black triangles), 25 °C. Data are provided in Supplementary Data file 1.

Detoxification of VX and Sarin was measured by an enzyme rescue assay as a function of time (see “Methods” section). The rate of VX- and Sarin-BChE reactivation was measured using the Ellman assay^16^. Four oximes demonstrated potent Sarin detoxification and VX- or Sarin-inhibited BChE reactivation (Fig. 1a). Known mono- and bis-quarternary oximes ChE reactivators, 2-PAM and HI-6, were employed as reference compounds (Table 1). To our knowledge, the unique non-quaternary chemical structures of these hit oximes (Fig. 1a) have never been reported as OP-inhibited ChE reactivators nor as OPNA detoxifiers. One oxime, 2-amidoxime 3-hydroxy pyridine (PCM-0212399) was reported previously as a direct detoxifier of a phenyl thiophosphonate analog of VX^17^. Two compounds are oximes with an unusual molecular structures based on benzaldoxime (PCM-0211955) and benzamidoxime (PCM-0211088) scaffolds. Interestingly, these two oximes were devoid of either the pyridinium or imidazolium ring that is present in most known oxime reactivators such as 2-PAM and CBIO (Supplementary Scheme 1). Dialkyl ketoximes (either aliphatic or alicyclic) have not been reported previously as detoxifiers of OPNAs, or as reactivators of OP-inhibited AChE or BChE.

**Table 1:** The kinetics of VX- and sarin-detoxification, and sarin-BChE and VX-BChE reactivation by HTS-identified oximes.

Compound name	Sarin detoxification *k*_obs_ × 10^3^ (min^−1^) [*t*_1/2_ (min)]	VX detoxification *k*_obs_ × 10^3^ (min^−1^) [*t*_1/2_ min]	Sarin-BChE reactivation *k*_obs_ × 10^3^ (min^−1^) [*t*_1/2 _min] % max react.	VX-BChE reactivation *k*_obs_ × 10^3^ (min^−1^) [*t*_1/2_ min] % max react.
PCM-0211955	71 [9.7]	n.a.	44 [15.7], >95	52, [13.3], 85
PCM-0212399	133 [5.2]	1.5, [450]	7.5, [92], 70	35, [19.7], 60
PCM-0211338	29 [23.7]	n.a.	2, [345], 40	3, [230], 30
PCM-0211088	11 [64.5]	n.a.	22 [31.4], >95	44, [15.7], 87
2-PAM	69, [10]^a^	9.1, [76]^b^	44, [16], 70	1.3, [528], 90
HI-6	58, [12]^a^	42, [16]^b^	32, [21.6], 94	14, [49], 80
Buffer	~3.5 [197]	0.1, [>6,900]	<5% react., 24 h	<5% react., 24 h

Detoxification of VX and sarin (1 µM) by oxime (0.5 mM), including 2-PAM and HI-6 as controls. Detoxification of sarin was measured with 1 µM sarin and 0.5 mM oxime, 20 mM phosphate, pH 8, 25 °C. In the reactivation experiments [VX] = 1.0 × 10^−6^ M and [sarin] = 3.0 × 10^−6^ M were used for BChE inhibition. HuBChE (2.0 × 10^−6^ M) was incubated with either VX or sarin until reaching a steady 90–95% BChE inhibition, before diluting the OP-BChE conjugate 100-fold into oxime or buffer solution, ([oxime] = 0.5 mM), 10 mM phosphate pH 8.0, 25 °C.

*n.a.* Not active

^a^Ref. ^26^ measured with sarin 1.5 mM and 15 µM oxime, 100 mM MOPS, pH 7.4, 25 °C.

^b^Ref. ^27^.

### Detoxification of VX and Sarin

We next focused on determining the kinetic parameters *k*_obs_ (min^−1^) and *t*_1/ 2_ (min) for the detoxification of VX and Sarin by the HTS-identified oximes (at 1 µM OPNA and 500 µM oxime), as well as initial rate constants (*k*_obs_ min^−1^) for reactivation of VX- and sarin-inhibited BChE (Table 1). The concentrations used for in vitro detoxification approximated blood levels of OPNA during intoxication (~1 µM) and the expected maximal concentration of oximes (100–500 µM) administered at relatively high doses during antidotal treatment of OP poisoning in vivo. The sole oxime which detoxified VX was PCM-0212399 (half-life of 7.5 h with complete VX degradation within 24 h) (Fig. 1b). The results were confirmed by a parallel experiment using LC/MS analysis (Supplementary Fig. 3. Data are provided in Supplementary Data file 2). These results likely indicated the formation of a benign moiety between VX and the PCM-0212399 amidoxime that could not re-inhibit BChE. (Fig. 1b). For higher fragment analysis resolution, the experiment was repeated at 1 mM VX with 1 mM amidoxime (Supplementary Fig. 4. Data are provided in Supplementary Data file 3). *O*-ethyl methylphosphonic acid (EMPA) was the main degradation product identified by LC/MS analysis during VX degradation. A second product was a 5-amino isoxazole ring fused to the pyridine ring, and is likely the product of an intramolecular cyclization of phosphoryl-amidoxime intermediate and EMPA (Supplementary Scheme S3). A similar isoxazole structure and *O*-ethyl phenylphosphonic acid formed during the degradation of a phenyl analog of VX has been described^17^.

The pyridine-amidoxime (PCM-0212399), benzaldoxime (PCM-0211955), benzamidoxime (PCM-0211088), and the ketoxime (PCM-0211338) (Fig. 1a) demonstrated different rapid rates of Sarin detoxification (*k*_obs_ = 133, 71, 11 and 29 × 10^−3^ min^−1^), respectively (Fig. 1c and Table 1). Detoxification of Sarin by all oximes was complete within 2 h. The rates of oxime-elicited Sarin detoxification were 3–38-fold faster than the rate of spontaneous hydrolysis of Sarin in 20 mM phosphate buffer pH 8, 25 °C (*k*_obs_= 3.5 × 10^−3^ min^−1^) (Fig. 1c and Table 1). PCM-0211338 is remarkable as this family of nucleophiles has not been previously described as detoxifying Sarin. Further, a close analog lacking the ketoxime moiety was completely inactive as a detoxifier, indicating that this part of the molecule is involved in the nucleophilic attack on Sarin. As far as we have been able to determine, all of the reported ChE reactivating oximes have been based mostly on aldoxime nucleophiles (R–CH=N–OH).

Pyridine-aldoximes, such as 2-PAM and HI-6, form phosphonyl-aldoxime (PhosAlox) intermediates when aldoximes react directly with an OP ester or while reactivating OP-inhibited ChE. The PhosAlox intermediate formed from the reaction of 2-pyridine aldoximes with Sarin has been determined to be relatively unstable, whereas the PhosAlox product (4-PPAM) of the reaction between 4-pyridine aldoxime and Sarin was relatively stable (*t*_1/2_ = 69 min)^18^. Interestingly, the hydrolysis of 4-PPAM generates a 4-cyano pyridine analog, thus diminishing the oxime level during either OP-ChE reactivation or OP detoxification. Critically, at high pH, the phosphoryl-ketoxime intermediates (PhosKox), produced by the reaction of ketoxime with organophosphoryl esters, are hydrolyzed via a different nucleophilic substitution pathway that regenerates the initial ketoxime and the corresponding alkyl-phosphoric acid^19^.

We first delineated the fragmentation of the ketoxime PCM-0211338 by LC/MS/MS analysis (Supplementary Fig. 5 and Supplementary Scheme 4). Then we analyzed the reaction products formed during the reaction of the unique piperidyl-ketoxime PCM-0211338 (1 mM) with sarin (1 mM) using LC/MS/MS fragmentation. The results suggested the formation of a PhosKox adduct (Supplementary Fig. 6 and Supplementary Scheme 5). Excitingly, eventual hydrolysis of the PhosKox would yield back the ketoxime (which in that case would act as a detoxification catalyst) and the corresponding *O*-isopropyl methylphosphonic acid (IMPA). We observed LC/MS/MS evidence for the formation of IMPA during PCM-211338 “catalyzed” hydrolysis of Sarin (Supplementary Figs. 5, 6 and Supplementary Scheme 5). The amount of the phosphonyl-ketoxime intermediate reached a maximum within the first hour of the reaction between Sarin (1 mM) and PCM-0211338 (1 mM), and then disappeared over the ensuing 24 h (Supplementary Fig. 7. Data are provided in Supplementary Data File 4). We also observed a parallel time-dependent decrease in Sarin and increase in IMPA concentrations (Supplementary Fig. 7). We expected the hydrolytic decomposition of the PhosKox adduct to regenerate the starting ketoxime as predicted by Van Hooidonk et al.^19^. The catalytic nature of this ketoxime will be the focus of a separate paper, but the results reported herein open the door to small molecule catalytic detoxifiers.

### Reactivation of VX- and Sarin-inhibited BChE

**Fig. 2:**
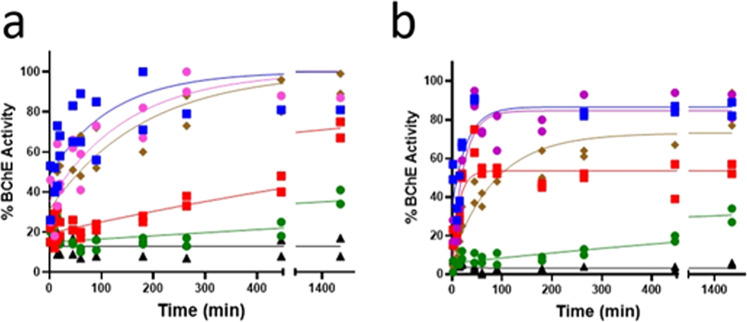
Reactivation of Sarin- and VX-inhibited BChE. Time-course of reactivation of **a** Sarin-inhibited BChE and **b** VX-inhibited BChE by PCM-0211088 (purple circles), PCM-0211955 (blue squares) PCM-0212399 (red squares), PCM-0211338 (green circles), and HI-6 (brown diamonds). [Oxime], 0.5 mM. VX-BChE reactivation was performed following inhibition by [VX] = 10^−6^ M, [BChE] - 2 × 10^−6^ M. Sarin-BChE reactivation was performed following inhibition by [Sarin] 3 × 10^−6^ M, [BChE] 2.5 × 10^−6^ M, [oxime] 0.5 mM, 10 mM phosphate pH = 8.0 (black triangles), 25 °C. Data are provided in Supplementary Data file 1.

The most potent reactivators of Sarin-inhibited BChE were PCM-0211955 PCM-0212399 and PCM-0211088 (*k*_obs_ = 44 × 10^−3^, 7.5 × 10^−3^, and 22 × 10^−3^ min^−1^, 0.5 mM, respectively, Table 1), with 70–95% reactivation within 1–1.5 hours (Fig. 2a and Table 1). Notably, two of these potent Sarin-Inhibited BChE reactivators (PCM-0211955 and PCM-0211088), have no pyridine ring or other nitrogen-containing heterocycle in their chemical structure (Fig. 1a). Each of the HTS-identified oximes (PCM-0211955, PCM-0212399, and PCM-0211088) reactivated VX-BChE 3.7-, 2.5-, 3.1-fold faster than HI-6, and 27–40-fold faster than 2-PAM, based on initial rates k_obs_ (min^−1^), 52 × 10^−3^, 35 × 10^−3^, 44 × 10^−3^, compared to 1.3 × 10^−3^, respectively, with 60–87% maximal reactivation within 24 hours (Fig. 2b and Table 1). The potentially catalytic ketoxime (PCM-0211338), was a slower reactivator than the other three hit oximes (*k*_obs_ 3 × 10^−3^ min^−1^), but was still threefold faster than 2-PAM, with maximal reactivation of 30% within 24 h (Fig. 2b and Table 1).

The ketoxime PCM-0211338, was a less potent reactivator of Sarin- and \vx-inhibited BChE with 40 and 30% maximal reactivation levels, respectively, (Fig. 2a, b and Table 1). Based on our LC/MS analysis and exact mass measurement of MS/MS fragments formed from PhosKox formed in the reaction between PCM-0211338 and Sarin (LC/MS/MS data in Supplementary Information Fig. 6, Supplementary Schemes 4 and 5), we have surmised that PCM-0211338 displaced the organophosphonyl moiety from the BChE active site by forming a PhosKox intermediate. The regeneration of intact ketoxime following the hydrolysis of this PhosKox intermediate was predicted by Van Hooidonk^19^.

### Reactivation of VX- and Sarin-inhibited BChE by alkyne oxime analogs

We have recently reported that oximes that have been coupled to polymer backbones via click chemistry can reactivate OP-inhibited cholinesterases^15^. We were therefore interested in whether a family of newly synthesized clickable alkyne-oximes (chemical structures are shown in Supplementary Scheme 6), derived from the HTS-identified leads, would also reactivate the inhibited enzyme. Alkyne analogs were synthesized and then tested for their reactivation potency toward VX- and sarin-inhibited BChE and direct detoxification of VX and sarin. Two of the alkyne analogs PCM-0214534 and PCM-0214528 were particularly potent for both VX- and sarin-inhibited BChE at 1 mM, pH 8, 25 °C. The *k*_obs_ for VX-inhibited BChE reactivation were 34 × 10^−3^ and 31 × 10^−3^ min^−1^, and the k_obs_ for sarin-inhibited BChE were 1.9 × 10^−3^ and 21 × 10^−3 ^min^−1^, respectively (Supplementary Table 1 and Supplementary Fig. 8). The alkyne analog PCM-0214528 derived from the hit oxime PCM-0211088 contained a 4-*O*-propargyl linker on the benzamidoxime aromatic ring that likely caused marginal steric hindrance to the amidoxime nucleophile during reactivation of OP-BChE. The rate of PCM-0214528-induced reactivation of VX-inhibited BChE, *k*_obs_ = 31 × 10^−3^ min^−1^ was 77-fold faster than that measured for its 5-propargyl analog PCM-0214517 (0.4 min^−1^). The alkyne analog PCM-0214534 derived from PCM-0212399 had a pentyl linker for binding the *N*-methyl *N*-propargyl amino group to 3-hydroxy 2-amidoxime pyridine ring (Supplementary Scheme 6). The *k*_obs_ values for the reactivation of VX- and sarin-inhibited BChE by PCM-0214534 (1 mM) were 34 × 10^−3^ and 1.9 × 10^−3^ min^−1^, respectively (Supplementary Table 1 and Supplementary Fig. 8). These k_obs_ values were similar to the rates measured for the pyridine-amidoxime PCM-0212399, but the rates of VX- or Sarin-inhibited BChE reactivation by the alkyne analog PCM-0214535 were significantly slower.

### The pH dependence of the rate of Sarin and VX detoxification by oximes

The only oxime that we discovered that could detoxify VX was PCM-0212399. This oxime exhibited faster kinetics at pH 8 (*t*_1/2_ ~ 10 h) than 7 (*t*_1/2_ ~ 20 h), with marginal VX hydrolysis generated at pH 6. All oximes and their alkyne analogs had rapid rates of sarin detoxification at pH 8 that gradually decreased at pH 7 and 6 (Supplementary Table 2). The most active sarin detoxifiers were PCM-0212399 and PCM-0211955 (1 mM) with *t*_1/2_ of 5 and 11 min at pH 7 and 1 and 3 min at pH 8, respectively. This pH dependence clearly indicates that the nucleophile species that directly attack the electrophilic OPNAs are the oximate anions.

### The pH dependence of reactivation rate of VX- and Sarin-inhibited BChE by oximes

Unexpectedly, all of the HTS-identified oximes displayed the fastest rate of reactivation at pH 7 compared to pH 6 and 8 (Supplementary Table 3). 2-PAM reactivated VX-BChE at much lower rates than the hit oximes at all pH values, and its reactivation rate was maximal at pH 8.7. In contrast to the pH-dependent reaction rate of VX-BChE reactivation, PCM-0211955, PCM-0212399, and PCM-0211338 had faster reactivation with Sarin-inhibited BChE at pH 8 compared to pH 7 and 6 (Supplementary Table 4). PCM-0211088, however, had a faster rate of reactivation of Sarin-BChE at pH 7 versus pH 8.

### Acute toxicity of oximes in mice

**Fig. 3:**
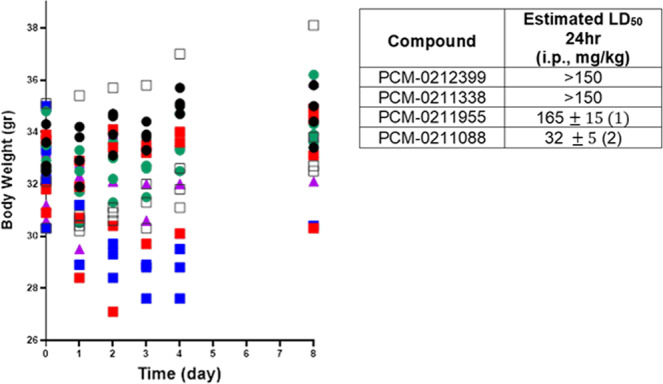
Time-course of change in daily body weight of mice and acute toxicity of oximes. Time-course of change in daily body weight of mice and acute toxicity LD_50_ values. Mice were injected with oximes at specified doses (i.p., *n* = 4). PCM-0211338 (green circle), PCM-0212399 (red square) and PCM-0211955 (blue squares) were administered at 150 mg/kg i.p. PCM-0211088 (purple triangles) was administered at 25 mg/kg i.p. DMSO (black open squares) was administered at 0.1 ml i.p. as solvent control. Sham animals (black circles) were not treated. Ordinary one-way ANOVA comparing body weights of oxime-treated mice to DMSO control (black open squares) display a significant difference for PCM-0211955 treated mice (blue squares) (*p* < 0.001). Data are provided in Supplementary Data file 1. (1) At a dose of 150 mg/kg (*n* = 4) mild toxic signs of PCM-0211955 were observed within 4–6 h post-injection. After 24 h one out of four animals died. At day 5 two more animals died. The curve of daily weight change of surviving animals indicated early toxic signs on day 1. (2) The LD_50_ of PCM-0211088 (24 h) and PCM-0211955 (48 h) was determined by the up and down method (see “Methods” section).

Acute toxicity testing was performed, including clinical monitoring and body weight over 8 days on surviving animals. PCM-0212399 and PCM-0211338 displayed no toxicity (150 mg/kg i.p.). Mild toxicity was seen for PCM-0211955, with a significant loss of weight and partial recovery over 8 days (Fig. 3). Ordinary one-way ANOVA comparing body weights of oxime-treated mice to DMSO control (black open squares) displayed a significant difference for PCM-0211955 treated mice (blue squares) (*p* < 0.001) (Fig. 3). However, 1/4 animals died at 24 h, with LD_50_ at 48 h determined to be 165 mg/kg (Table insert in Fig. 3). PCM-0211088 was lethal at 150 mg/kg with LD_50_ of 32 mg/kg (within 24 hours) (Table insert in Fig. 3). The toxic signs for PCM-0211088 (25 mg/kg i.p) were typical of cholinergic toxicity to the central nervous system (convulsions, apnea). Interestingly, literature LD_50_ values for known quaternary oximes 2-PAM and HI-6 are 163 ± 4 and 638 ± 42 mg/kg (ip), respectively, in mice^20^. Non-quaternary oximes PCM-0212399, PCM-0211338, PCM-0211955 could penetrate the BBB thus their measured toxicity is low compared to known quaternary oximes.

## Conclusions

Two HTS campaigns with >150,000 small molecules each were performed using fluorogenic EMP-MeCyC as a surrogate of OPNAs for the discovery of new OPNA detoxifiers and OPNA-BChE reactivators. Validation of the HTS-discovered compounds resulted in the identification of four new oximes for detoxification of VX and Sarin, and the reactivation of VX- and Sarin-inhibited BChE. These oximes had unique chemical structures in which the benzaldoxime (PCM-0211955) and benzamidoxime (PCM-0211088) were devoid of any nitrogen atom in the oxime-carrying aromatic ring, and also included a pyridine amidoxime (PCM-0212399) and a dialkyl ketoxime (PCM-0211338). These chemical structures have not been reported previously as OPNA-BChE reactivators nor as OPNA scavengers. All discovered non-quaternary oximes reactivated VX- and Sarin-inhibited BChE faster than quaternary 2-PAM. It should be emphasized that all the oximes discovered in this study were non-quaternary oximes, and PCM-0211338 has a unique ketoxime moiety. The ketoxime formed a Phosphonyl-Oxime intermediate when reacted with Sarin that, upon pH-dependent hydrolysis, will regenerate the ketoxime, thus rendering it a catalytic scavenger. Designing future molecules with pKa values aligned with the physiological environment is expected to yield better OPNA-detoxifying small molecule catalysts. Based on the reactivation potency in vitro as well as expected permeability via the BBB, based on calculated logD values and their relatively lower toxicity in mice in vivo, the newly discovered oximes could be of interest for both antidotal therapy against poisoning by OP nerve agents, or as adjunct drugs with BChE, converting the stoichiometric OP sequester BChE into a pseudo-catalytic bio-scavenger. Two alkyne-oximes, PCM-0214528 and PCM-0214534, preserved the reactivation and detoxification potency of their parental HTS-discovered oxime hits and are starting points for biocompatible azido-polymer conjugates useful for extended in vivo lifetime.

## Methods

### Materials

Sodium phosphate salts, acetylthiocholine, DTNB, Bovine serum albumin, 2-PAM, and HI-6 were purchased from Merck (Sigma, Israel). *O*-ethyl methyl phosphonate methyl cyano-coumarin (EMP-MeCyC) was synthesized as described previously^21^. Resorufin pivalate was synthesized according to ref. ^22^. The HTS hit 3-hydroxy 2-amidoxime pyridine (PCM-0212399, Fig. 1a) was synthesized according to ref. ^17^. A nucleophile-focused library of 1348 nucleophilic compounds was composed of 1298 aldoximes, hydroxamates, alkylhydroxamtes, ketoximes, and amidoximes purchased from Enamine, Kiev. Ukraine. The following 18 quaternary aryl-imidazolium aldoximes (ImOx’s), ProImOxmCl, ProImOxpCl, ButImOxmCl, ButImOxpCl, BeImOxmCl, BeImOxpCl, pClpClImOx, pClmClImOx, mClmClImOx. pCH3pClImOx, pCH3mClImOx, mCH3pClImOx, mCH3mClImOx, BeImOx, pClImOx, mClImOx, pCH3ImOx and mCH3ImOx, together with 3 Cinchona-derived quinuclidinium-quinoline oximes Ox(C1), OxCH3I (C2) and OxBnBr (C3)—were kindly provided by Z. Kovarik, IMI, Zagreb, Croatia. The following 7 substituted benzohydroxamic acids (BHA), HA-1, HA-2, HA-2, MeBHA-2, MeBHA-3, BuBHA-4, and BuBHA as well as 13 Pyridinium aldoxime N-substituted by alkyl amides and alkyl amino acids, IL-1, IL-2, IL-3, IL-Ala-1, IL-Ala-2, IL-Ala-3, IL-Phe-1, IL-Phe-2, IL-Phe-3, IL-PheO-1, IL-PheO-2, IL-PheO-3, and IL-Phe-acid, were kindly provided by Y. Karpichev, Tallinn University of Technology, Tallinn, Estonia. The following nine bis-quarternary pyridinium oximes: K027, K048, K203, K865, K866, K867, K868, K869, and K870 were kindly provided by Kamil Musilek, University of Hradec Kralove, Hradec Kralove, Czech Republic. The following alkyne analogs of the hit oximes were custom synthesized at Enamine, Kiev, Ukraine. PCM-0214580, PCM-0214579, PCM-0214535, PCM-0214534, PCM-0214533 and PCM-0214518 (Supplementary Scheme S6).

VX and sarin were synthesized at IIBR according to previously published procedures with utmost safety precautions. Purified human serum BChE was kindly provided by O. Lockridge, Omaha Medical Center, Omaha, Nebraska, USA. Purified recombinant Human AChE was kindly provided by the Department of Biotechnology, IIBR, Israel.

### Screening libraries

The HTS screening campaigns for detoxification and reactivation were conducted with the small molecule compound library of the Israel National Center for Personalized Medicine at the Weizmann Institute of Science. The library is composed of commercial sets from ChemDiv (Diversity), Chembridge (DiversetCl), Enamine (Drug-like Set, and Nucleophiles), Maybridge (Hitfinder), Microsource (Spectrum), Selleck (Bioactives), Prestwick (Known Drugs), and Sigma (LOPAC). Additional contributions from academic collections (listed above) were registered and integrated into the screening deck prior to conducting the screens.

### HTS equipment

High-throughput screens, counter screens, orthogonal screens and hit validation experiments were performed using the HTS equipment: Echo555 Acoustic transfer system (Labcyte, Germany), MultiDrop 384 (Thermo Scientific), Washer Dispenser II (GNF, San Diego, CA, USA), EL406 Microplate Washer Dispenser (BioTek, Winooski, VT, USA), Bravo Automated Liquid Handling (Agilent, Santa Clara, CA, USA). Fluorescence/absorbance signals were measured by luminescence module of PheraStar FS plate reader (BMG Labtech, Ortenberg, Germany). 1536-well plates were from Nunc (#264711), and 384-well plates from Greiner (#781162).

### Synthesis

The synthetic pathway for preparing the O-propargyl alkyne benzamido oxime analogs PCM-0214528 (4a) PCM-0214517 (4b), PCM-0214522 (4c) are shown in the Supplementary Information (Supplementary Scheme 2).

### HTS fluorescent detoxification assay

The assay buffer (AB) was 10 mM phosphate buffer, pH = 7.9. 1536-well plates were pre-plated with compounds and controls (480 nl/well, final 600 µM) using the Echo transfer system. The nucleophilic compound Benzhydroxamic acid (BHA) was used as a standard. 10 mM DMSO stocks of either EMP- MeCyC (working solution, neutral control) or MeCyC-OH (positive control) were diluted 2,000-fold in AB to 5 µM (final concentration of 2.5 µM). 8 µl of the solutions were added to appropriate wells of 1536-well plates and 380/430 fluorescence emission was read twice: immediately (time 0 h) and after 3 h incubation using a PheraStar plate reader. Data analysis was as follows: calculation of average – standard deviation (SD) of fluorescence at time 0 h; calculation of average fluorescence at time 3 h; calculation of the average ratio of fluorescence at 3 h/0 h. Compounds that were able to increase fluorescence signal at least 70% above BHA-induced fluorescence (standard) were selected for validation. In order to filter out false-positive compounds, hits were also subjected to a counter assay, where MeCyC-OH was used instead of EMP-MeCyC.

### HTS fluorescent reactivation assay

The assay buffers were: Enzyme buffer (EB), 10 mM Na Phosphate pH 7.6; Substrate buffer (SB), 10 mM Na phosphate pH 6.7, 0.01% BSA. 1536-well plates were pre-plated with compounds and controls (40 nl/well, final 100 µM in 4 µl) using an Echo transfer system. 2-PAM was used as a reference oxime. Either BChE (non-inhibited enzyme, positive control) or a mixture of BChE + EMP-MeCyC (inhibited enzyme, optimized to ~85% enzyme inhibition; working solution, neutral control) was diluted in EB under sterile conditions (BChE stock 25 µM in EB, EMP-MeCyC 10 mM stock in DMSO; final BChE concentration of 1.6 µM). These solutions were incubated for 30 min at room temperature and then diluted 50-fold in EB to 32 nM enzyme solution. 4 µl of each solution were added to appropriate wells of 1536-well plates and incubated in the presence of tested compounds for a further one hour at room temperature (reactivation reaction). Resorufin pivalate, 10 mM stock, was diluted 2000-fold in SB to 5 µM, and 4 µl were added to each assay well (final concentration of the enzyme and EMP-MeCyC was 16 nM; RP 2.5 µM). After additional incubation for one hour at room temperature in the dark, the 540/590 fluorescence signal was read. Compounds that yielded an equivalent or larger increase in fluorescence signal to that obtained for 2-PAM were selected for follow-up validation in a double dilution assay. Using 2-PAM as a selection gate minimized false hits from direct oximolysis of the substrate by the nucleophiles. BChE and BChE + EMP-MeCyC were diluted to 6.4 µM, incubated for 30 min, then diluted 50-fold to a concentration of 128 nM. Next, the solutions were added to 384-well plates and diluted again 4-fold to 32 nM BChE. All following steps were as above.

The suitability and reliability of the detoxification and reactivation HTS assays were evaluated by determining the screening window coefficient parameter *Z*-prime (*Z*′). *Z*′ is reflective of both the assay signal dynamic range and the data variation associated with signal readouts. We measured *Z*′ value as an assay quality assessment and to demonstrate that the fluorescent readout in our HTS assays was large enough to warrant reliability and reproducibility^23^. *Z*′ was calculated by the following equation:

$$Z^{\prime}=1{-}3({\sigma }_{{\rm{p}}}+{\sigma }_{{\rm{n}}})/|{\mu }_{{\rm{p}}}{-}{\mu }_{{\rm{n}}}|$$

where *σ*_p_ and *σ*_n_ are the standard deviations of the mean values *µ*_p_ and *µ*_n_ of the positive (p) and negative (n) control signals. The mean and standard deviation of the positive and negative controls were based on 32 repeated readouts of these controls in 1536-well plate. Usually, *Z*′ values of 0.5–0.9 reflect highly reliable HTS assays.

### HTS absorbance-based Ellman assay

The assay buffers EB and SB were as described above. 384-well plates were pre-plated with potential hit compounds discovered in the fluorescent HTS described above. BChE and BChE + EMP-MeCyC were diluted in EB to 1.6 µM and incubated for 30 min at room temperature. Then, the solutions were diluted 200-fold in EB to 8 nM, dispensed to appropriate wells, and incubated for a further one hour at room temperature. Then, the recovered activity of BChE was measured by the Ellman absorbance assay^16^ as follows: 60 µl of SB were added to each well, following by the addition of 15 µl of DNTB (stock 5 mM, final 0.75 mM and substrate acetylthiocholine (ATC), stock 7.27 mM, final 1.1 mM). Absorbance was measured continually at 412 nm (OD_412_) over time. In parallel, the absorbance at 412 nm in wells containing all Ellman reagents, without the enzyme, was measured in order to evaluate the rate of direct oximolysis of ATC.

### Detoxification kinetics using an “enzyme rescue assay”

Prior to starting the OPNA degradation reaction, a 96-well plate was filled with 120 µl of 50 mM sodium phosphate buffer at pH 7.4, 30 µl of DTNB (final 0.75 mM), and 10 µl of BChE (final 1 × 10^−9^ M). The OPNA degradation reaction (1 µM of either VX or sarin) was incubated with the screened oximes (0.5 mM) at pH 8 at room temperature.

At specified time intervals, samples of 10 µl of the degradation reaction mixture were drawn and added to the 96-well plate (diluted 20-fold in 200 µl), that contained BChE (10^−9^ M) in 50 mM phosphate buffer pH 7.4 with DTNB, and incubated for 3 min at room temperature. The 3-min inhibition period was determined independently for obtaining 90–95% inhibition of BChE (10-9 M) by intact OPNA (5 × 10^−8^M sarin). Finally, 10 µl of the substrate acetylthiocholine (ATC) solution was added (final ATC concentration was 1.1 mM in 200 µl total volume) to start the Ellman enzymatic activity reaction (increase in OD405nm 1 min readout time). The Ellman assay for BChE activity was performed at pH 7.4 that keeps ATC stable and reactive. It should be emphasized that in the OP degradation assay, BChE serves only for monitoring the residual potency of the OPNA as enzyme inhibitor during its degradation by the oximes at pH 8 (using pH 8 for the reason described above) and the Ellman assay at which ATC is more stable and reactive, was performed at pH 7.4. The decrease in BChE inhibition caused by progressive degradation of VX and sarin with time is presented in Fig. 1b, c, respectively. Absorbance was read at 405 nm using a Tecan, Infinity F200 M spectrophotometer.

### Reactivation of VX-BChE and sarin-BChE with HTS-selected Oximes

The reactivation reaction was performed in two stages. First, BChE at a concentrated solution (2.5 × 10^−6^ M) was inhibited by the OPNA (5 × 10^−6^ M sarin) in 20 mM phosphate buffer pH 8 containing 0.01% BSA—to provide about 90–95% steady-state inhibition within 30–60 min. Secondly, the OP-BChE conjugate was diluted 4-fold into 20 mM phosphate buffer pH 8 containing 0.01% BSA and then further diluted 25× fold into either the same buffer (pH 8) or buffer containing the screened oxime (0.5 mM). In parallel, free BChE was diluted 4-fold and then 25-fold in pH 8 to serve as a control uninhibited enzyme (*E*_*0*_). The total dilution of the concentrated OP-BChE conjugates was 100-fold in buffer or oxime-containing buffer. The 4-fold and 25-fold consecutive dilutions were done to handle solutions volume scale in a more operationally convenient manner. The reactivation reaction mixture, as well as the *E*_0_ solution, were sampled (10 µl) at the same specified time intervals and diluted into 160 µl of 50 mM phosphate pH 7.4 that contained DTNB. Finally, 30 µl of ATC was added to start the Ellman activity assay for a 1 min readout time. It should be noted that the activity of free BChE (*E*_0_) was decreased by about 20–25% after 24 h at room temperature in 20 mM phosphate 0.01% BSA pH 8, however, the regained BChE activity during reactivation was compared to the corresponding *E*_*0*_ value measured at the same specified time point.

### Toxicology

Adult male Hsd:ICR mice (Envigo, Rehovot, Israel), weighing 25–30 g, were housed 10 per plastic cage with bedding, under controlled environment at 21 °C ± 2 °C and a 12-h light/dark cycle with lights on at 7 am. The mice were acclimatized for 1 week before the experiment. Food and water were available ad libitum.

All the procedures involving mice were approved by and conducted in accordance with the institutional Animal Care and Use Committee at the Israel Institute for Biological Research (protocol-M-21-20, Israel Institute for Biological Research), and are also in strict accordance with the Guide for the Care and Use of Laboratory Animals (National Academies Press, Washington DC, 2011).

All oximes were dissolved in DMSO at 45 mg/ml. A group of four mice weighing 25–30 g was initially treated with each oxime at a dose of 150 mg/kg by injecting ~100 µl of the oxime solution intraperitoneally (i.p.). Toxic signs were monitored for 8 days, and the mice were weighed each day. Two of the oximes PCM-0211955 and PCM-0211088 that displayed toxicity at 150 mg/kg (i.p.) were estimated for their LD_50_ using the up and down toxicity method^24^.

### Statistics and reproducibility

#### HTS assays

The suitability and reliability of the detoxification and reactivation HTS assays were evaluated by determining the screening window coefficient parameter *Z*-prime (Z′) as defined above in the HTS methods.

#### Detoxification and reactivations kinetics

All kinetics of Sarin and VX detoxification as well as reactivation of VX- and Sarin-inhibited BChE were performed by three repeats of each experiment (*n* = 3). The symbols  represent individual values (*n* = 3). The kinetic parameters of detoxification in Fig. 1b, c (*k*_obs_ and *t*_1/2_) were calculated from a linear transformation of the data to Ln[%BChE Inhibition] vs. time. The kinetic parameters of reactivation (*k*_obs_, *t*_1/2_) in Fig. 2a, b were calculated from a non-linear fit to the mean values at each time point.

#### Toxicity in vivo

The time-course of change in mice body weight over 8 days after treatment with four oximes are presented in Fig. 3 as individual points (*n* = 4). Ordinary one-way ANOVA comparing body weight of oxime-treated to DMSO treated mice (control) was performed over a period of 8 days. All statistical analyses were performed with GraphPad Prism software.

### Reporting summary

Further information on research design is available in the Nature Research Reporting Summary linked to this article.

## Supplementary information

Supplementary Information

Description of Additional Supplementary Files

Supplementary Data 1

Supplementary Data 2

Supplementary Data 3

Supplementary Data 4

Reporting Summary

## Data Availability

The data sets for main Figs. 1, 2, 3, and Supplementary Figs. 3, 4, 7 generated during the current study are available from https://figshare.com/articles/dataset/Data_files/14176559 or from the corresponding author on request^25^.
