# Supplementary material for: Non-quaternary oximes detoxify nerve agents and reactivate nerve agent-inhibited human butyrylcholinesterase
Source: Commun Biol. 2021 May 14;4:573. doi: 10.1038/s42003-021-02061-w (PMC8121814; doi:10.1038/s42003-021-02061-w)
Supplement: Supplementary file 1 — Supplementary Information [file 42003_2021_2061_MOESM1_ESM.pdf]

Communications Biology Revised Manuscript No. **COMMSBIO-20-2893A**

Supplementary Information

Non-Quaternary Oximes detoxify nerve agents and reactivate nerve agent-inhibited Human Butyrylcholinesterase

Gabriel Amitai[(1)], Alexander Plotnikov [(1)], Shira Chapman [(2)], Shlomi Lazar [(2)] , Rellie Gez [(2)], Dan Loewenthal[(3)], Khriesto Shurrush[(1)], Galit Cohen [(1)], Leonardo J. Solmesky[(1)], Haim Barr [(1)] and Alan J. Russell[(4)]

(1) The Wohl Drug Discovery Institute, Grand Israel National Center for Personalized Medicine (G-INCPM), The Weizmann Institute of Science, Rehovot 7600001, Israel

(2) Department of Pharmacology, Israel Institute for Biological Research (IIBR), PO Box 19, Ness Ziona 7045000, Israel

(3) Department of Analytical Chemistry, IIBR, PO Box 19, Ness Ziona, Israel

(4) Department of Chemical Engineering, Carnegie Mellon University, Pittsburgh, PA. 15213, USA

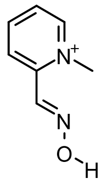

2-PAM

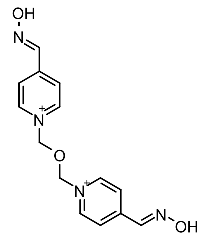

Toxogonin

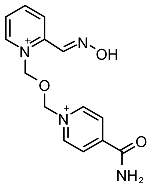

HI-6

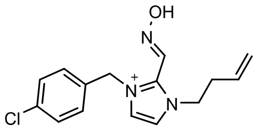

CBIO

Supplementary Scheme 1: Chemical structure of quaternary oximes known as reactivators of OP-AChE and OP-BChE (2-PAM, Toxogonin, HI-6, 1-(p-chlorobenzyl) 3-(but-1-enyl) 2-imidazolium aldoxime CBIO)

IUPAC chemical names of Oximes (structures of HTS discovered oximes are shown in the text in Figure 1A):

2-PAM 2-[(E)-(hydroxyimino)methyl]-1-methylpyridin-1-ium

HI-6 4-carbamoyl-1-[({2-[(E)-(hydroxyimino)methyl]pyridin-1-ium-1-yl}methoxy)methyl]pyridin-1-ium dichloride

Toxogonin 1,1'-[oxybis(methylene)]bis{4-[(E)-(hydroxyimino)methyl]pyridinium}upp dichloride

CBIO: (E)-1-(but-3-en-1-yl)-3-(4-chlorobenzyl)-2-((hydroxyimino)methyl)-1H-imidazol-3-ium chloride

PCM-0212399 (E)-N',3-dihydroxypyridine-2-carboximidamide

PCM-0211955 3-bromo-2-[(E)-(hydroxyimino)methyl]phenol

PCM-0211338 (8E)-6-benzyl-8-(hydroxyimino)-5H,6H,7H,8H-pyrido[4,3-d]pyrimidin-2-ol

PCM-0211088 (Z)-2-(benzyloxy)-N'-hydroxybenzene-1-carboximidamide

Supplementary Note 1*:* High Throughput Screening for hydrolysis of fluorogenic EMP-MeCyC (Detoxification screen)

In an initial screen of 152,304 compounds in 1536-well plates we used both non-focused and nucleophile-focused compound libraries in a carefully designed workflow (**Supplementary Figure 1A**). The EMP-MeCyC hydrolysis assay was determined to have a Z' (see Z' definition in Methods) of approximately 0.7, indicating that the assay was robust and dependable for the identification of potential nerve agent detoxifiers. HTS hits were subjected to a counter screen, where MeCyC-OH was used instead of EMP-MeCyC in order to eliminate compounds that enhanced the fluorescence emission of the hydrolysis product MeCyC-OH. The primary hit rate in non-focused libraries was 0.02% (38/150,956x100), and in the nucleophile-focused library was 15.1%.(204/1,348x100) The top 19 detoxification hits of the fluorogenic detoxification assay with EMP-MeCyC were validated kinetically as detoxifiers of either Sarin or VX nerve agents (**Supplementary Figure 1B**). Sarin detoxifiers were selected based on k_{obs} > $20x10^3$ min^{-1}. Most detoxifiers discovered by the fluorogenic-based screen were also Sarin detoxifiers but only the oximes PCM-0211955, PCM-0211399, PCM-01211088 and PCM-0211338 displayed reactivation potency toward OPNA-BChE. The amidoxime hit PCM-0212399 also displayed detoxification activity toward VX (Figure 1b and 1c in the main text).

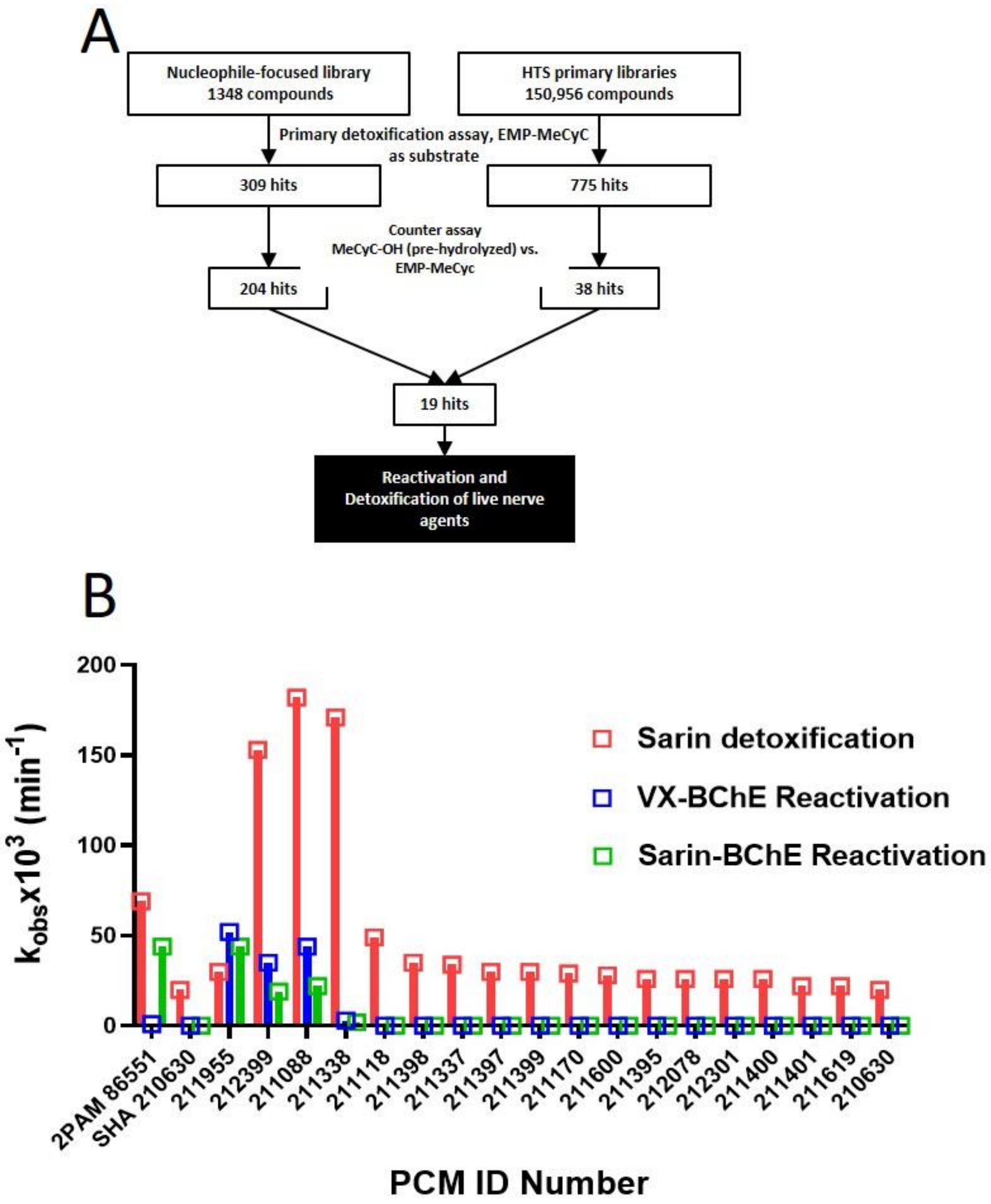

Supplementary Figure 1: Workflow of HTS for discovery of fluorogenic surrogate EMP-MeCyC detoxifiers and validation of hits with nerve agent detoxifiers. (A) HTS screens and filtering assays including counter assay with MeCyC-OH. (B) Nerve agents validation of 19 screening hits for Sarin detoxification, VX-BChE and Sarin-BChE reactivation with kinetic assays results (k_{obs}). 2PAM and Salicyl-hydroxamic acid (SHA) (further left) served as reference compounds for reactivation and detoxification, respectively.

Supplementary Note 2*: High throughput screening for the identification of reactivators of EMP-MeCyC-inhibited BChE: (Reactivation screen)*

We carefully designed a workflow for the HTS discovery of reactivators of EMP-MeCyC-inhibited BChE (**Supplementary Figure 2A**), in which the main screen for EMP-BChE reactivators was performed in 1536-well plates with Resorufin Pivaloate (RP) as a fluorogenic substrate of BChE. The screen employed 154,681 small molecules, and the Z’ (0.5) indicated that the assay was reliable. The HTS primary hits of EMP-BChE reactivation were subjected to a double dilution experiment (**Supplementary Figure 2A**) in which the primary hit rate after double dilution in non-focused libraries was found to be 0.07% (118/153,513x100), and in the nucleophile-focused library 4.6% (63/1348x100) (**Supplementary Figure 2A**). Next, we developed an independent assay for these hits using a different method in which the primary hits were subjected to an Ellman absorbance orthogonal/counter screen [1], where the readout was based on the absorbance of the thiocholine-DTNB adduct at 412 nm instead of fluorescence emission formed by the hydrolysis product of EMP-MeCyC (**Supplementary Figure 2A**). The 14 hits that were discovered in the HTS of EMP-BChE reactivation were validated kinetically as reactivators of either Sarin- or VX-inhibited BChE (**Supplementary Figure 2B**). The compound PCM-0212379 is a known imidazole oxime [2]. It displayed a high potency as reactivator of VX-BChE but low potency as reactivator of Sarin-BChE as well as detoxifier of Sarin. Bezamidoximes PCM-00213249 and PCM-0213250 are known nucleophiles [3] displaying high potency as Sarin detoxifiers but low reactivation potency.

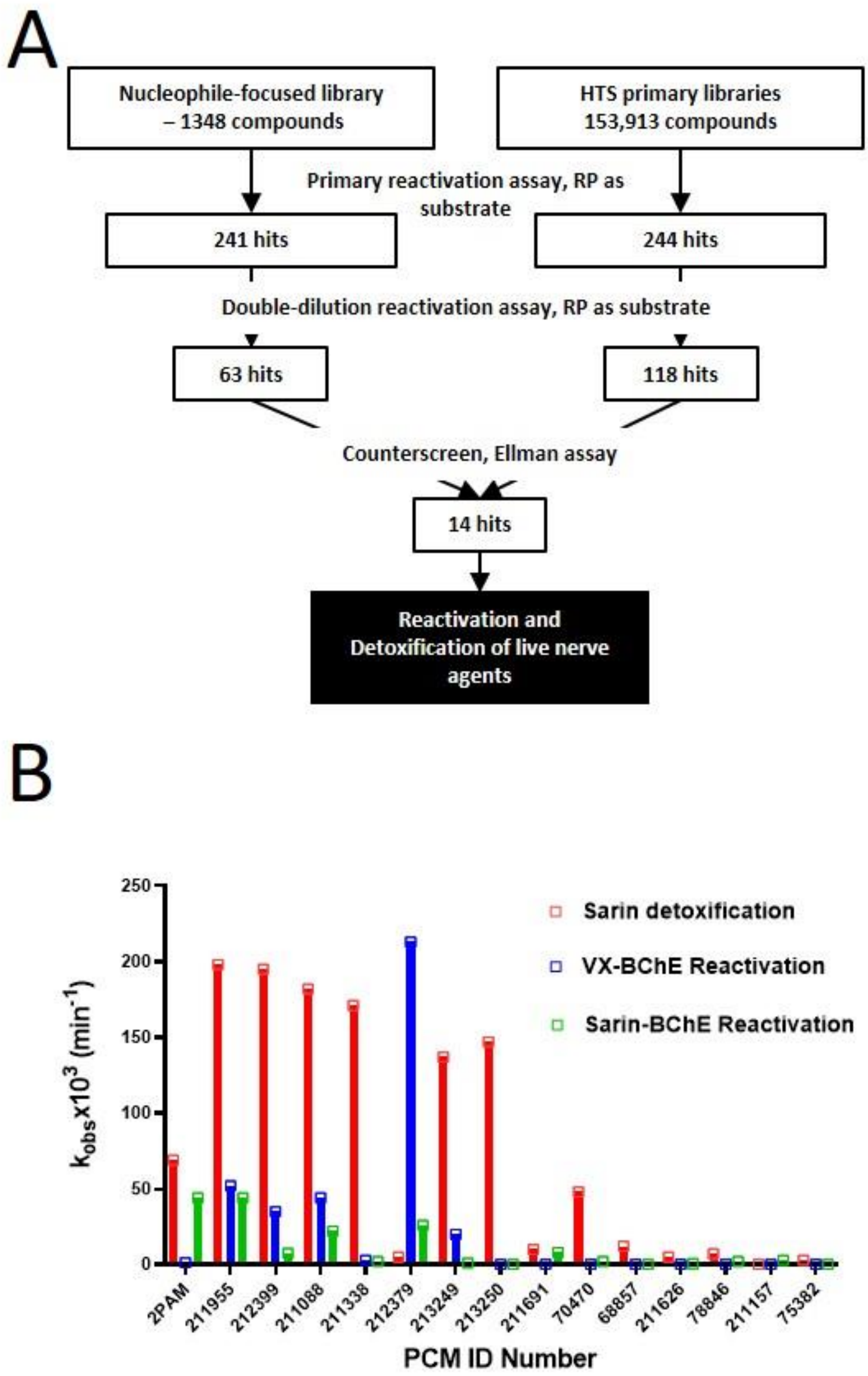

Supplementary Figure 2: Workflow of HTS for discovery of EMP-BChE reactivators and validation of hits with nerve agent-inhibited BChE

(A) - HTS screens and filtering assays including the Ellman absorbance assay [1]. (B)- Nerve agent validation of 14 screening hits for Sarin-

Detoxification, VX-BChE and Sarin-BChE rreactivation with kinetic assays results (k_{obs}). 2PAM (furthest left) served as reference oxime.

Supplementary Note 3*:* Synthesis of alkyne analogues of HTS-discovered oxime PCM-0211088

The synthetic pathway for preparing the O-propargyl substituted benzamido oxime analogues **4a-c** of the hit oxime PCM-0211088 is shown in **Supplementary Scheme 2**.

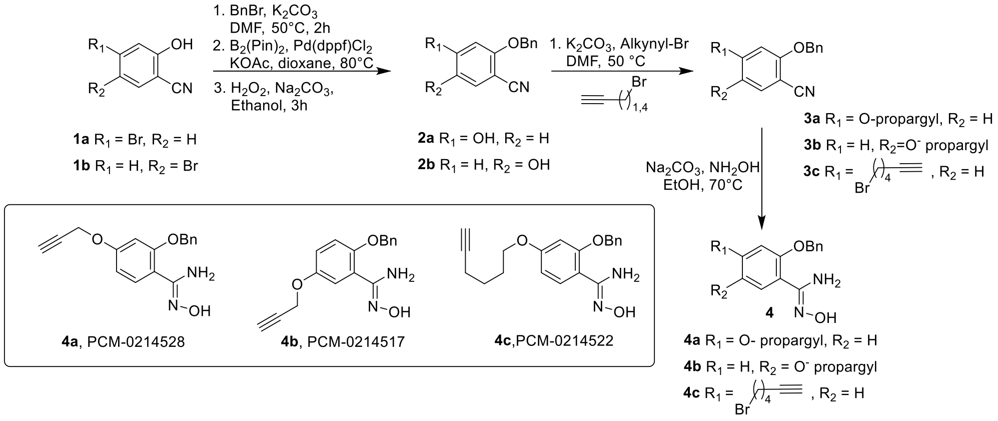

Supplementary Scheme 2: General synthetic pathway for preparing three alkyne analogues of the hit PCM-0212399 designated as **4a-c** in Supplementary Scheme 2**.**

The alkyne-substituted amidooximes **4a-c** (scheme 1) were prepared according to a representative procedure shown here for the preparation of **4a** alkyne analogue.

2-(benzyloxy)-4-hydroxybenzonitrile (2a): The corresponding 4-bromo-2-hydroxybenzonitrile(**1a)** (1.00 g, 5.05 mmol) was dissolved in 15ml of DMF, then added benzyl bromide (0.950 g, 5.6 mmol), K_2CO_3 (1.4 g, 10.1 mmol) and heated to 50°C for 12h, the reaction was cooled and poured into 50 mL of water and extracted 3 X EtOAc. The combined organic layers were washed 2 X water and 1 X brine, dried over Na_2SO_4 and concentrated. The compound was purified by flash chromatography using CombiFlash EZ Prep System gradient hexane to EtOAc, the desired compound eluted in 20% EtOAc. The fractions were concentrated to give 2-(benzyloxy)-4-bromobenzonitrile (1.24 g) as a white solid in 85 % yield.

In a sealed microwave vial the 2-(benzyloxy)-4-bromobenzonitrile (300 mg, 1.04 mmol), B_2Pin_2(317 mg, 1.258 mmol), KOAc (204 mg, 2.08 mmol) was then dissolved in 1,4-dioxane (10mL), the reaction was flushed with argon and then heated to 80ºC for 3h. The reaction was then filtered through a pad of celite and concentrated. The crude oil was dissolved in ethanol (10 mL) then Na_2CO_3 (441 mg, 4.16 mmol) was added, followed by dropwise addition of H_2O_2 30% (w/w) in water (0.43 mL, 0.16 mmol). The reaction was stirred for 12h. The reaction mixture was then poured into 20 mL water and acidified to pH=7 with HCl 1M and then extracted 3 X EtOAc. The combined organic layers were washed 1 X water and 1 X brine, dried over Na_2SO_4 and concentrated. The compound was purified by flash chromatography using CombiFlash EZ Prep System gradient hexane to EtOAc, the desired compound eluted in 60% EtOAc, to give **2a** (203 mg) in 85% yield.

2-(benzyloxy)-4-(prop-2-yn-1-yloxy)benzonitrile (3a): In a 25 mL round bottom flask **2a** (109 mg, .48 mmol) was dissolved in DMF (2 mL), then added K_2CO_3 (133.7 mg, 0.97 mmol) followed by propargyl bromide (108 uL, 0.97 mmol) and heated to 60°C for 2h. The reaction mixture was poured into 10 mL water and extracted by 3X EtOAc. The combined organic layers were washed 1 X water and 1 X brine, dried over Na_2SO_4 and concentrated. The compound was purified by flash chromatography using CombiFlash EZ Prep System gradient hexane to EtOAc, the desired compound **3** eluted in 40% EtOAc, to give **3a** (106 mg) in 83% yield.

2-(benzyloxy)-N'-hydroxy-4-(prop-2-yn-1-yloxy)benzimidamide (4a) The corresponding benzonitrile **3a** (44 mg, 0.16 mmol) was dissolved in EtOH (0.5 mL) and added to a solution of NH_2OH (47 mg, 0.67 mmol), Na_2CO_3 (74 mg 0.70 mmol) in water 2mL and the reaction mixture was transferred to a microwave vial, then the reaction vial was sealed and heated to 100ºC for 18h in the microwave oven. The reaction mixture was extracted 4X EtOAc, 2X DCM. The combined organic layers were dried on Na_2SO_4. The compound was purified by flash chromatography using CombiFlash EZ Prep System gradient DCM to EtOAc, the desired compound eluted in 40% EtOAc to give **4a** (9.5 mg) in 19% yield.

Supplementary Note 4: LC-ESI/MS/MS Experiments

4.1 Materials

Stock solutions of VX 10mM and Sarin 21.4mM in propylene glycol and phosphate buffer 20mM (pH=8.0 and pH=7.0) were prepared in the Department of Pharmacology (IIBR). The amidoxime hit PCM-0212399 was synthesized at the G-INCPM, WIS. The ketoxime PCM-0211338 purchased from Enamine, Kiev, Ukraine. Conversion of Sarin to dimethyl aminophenol ester derivative (Sarin-Der) was performed by the reaction of Sarin with excess of the reagent, 2-[(N.N-Dimethylamino) methyl] phenol (DMAMP). DMAMP, ammonium formate (MS-grade) and dimethyl sulfoxide (DMSO) were purchased from Sigma-Aldrich (St. Louis, MO, USA). Water and methanol (LC-grade) were purchased from Bio-Lab ltd (Israel).

4.2 LC-ESI/MS/MS Analysis

The following LC/MS system was used for all LC-ESI/MS/MS experiments. The LC-ESI\MS\MS system consists of an Alliance 2690 (Waters) LC coupled to an Ultima Triple Quadrupole (Waters) via an ESI source. The ESI source was operated in both positive and negative modes with a spray voltages of 2500V,-2500V respectively and source temperature of 120°C. Chromatographic separation was achieved using a reverse phase column (Gemini C18, $3\mu m$, 150mm, 2mm ID Phenomenex) eluted with eluents A (1 mM ammonium formate in 5% methanol/water) and B (1mM ammonium formate in methanol) with the following gradient program time[min]/B[%]: 0/0,5/95,8.5/95,8.51/0,16/100. Injection volume was 10µl.

4.3 Reaction of PCM-0212399 (0.5mM) with VX (1µM)

VX solution in propylene glycol (10mM) was diluted 10:990 in LC grade water (0.1mM) then 10µl were added to 965uL phosphate buffer 20mM pH=8 and then added 25µL of PCM-0212399 in DMSO (3mg/ml, 19.6mM, MW 153), for a final concentrations of 1µM VX and 0.5mM PCM-0212399. Control samples were prepared similarly with 1µM VX in phosphate buffer with 2.5% DMSO. At every time point, 10µl of the reaction sample/buffer control (triplicate) was analyzed for residual VX by LC-ESI/MS/MS(MRM 268->128, 268->86,

Collision energy of 17eV). The kinetic plot is shown in **Supplementary Figure 3**

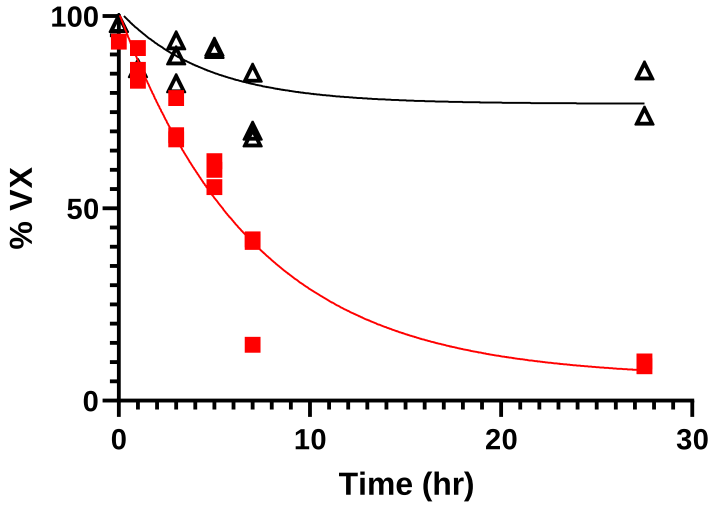

Supplementary Figure 3: Time Course of VX degradation measured by LC/MS analysis

Legend to Supplementary Figure 3: The amidoxime PCM-0212399 (final 0.5mM) was added (from 20mM stock in DMSO) to a VX solution (1µM) to 20mM phosphate pH 8, 25°C and samples were drawn at specified time intervals for LC-MS analysis (red squares). In parallel, VX solution in phosphate buffer containing 2.5% DMSO was sampled at the same time intervals (open black triangles). Residual % VX values analyzed by LC-MS are expressed as means with standard deviation (SD) (n=3) (experiment was preformed twice), K_{obs} = 0.14 hr^{-1} $t_{1/2}$ = 4.92 hrs

4.4 Reaction of PCM-0212399 (1mM) with VX (1mM)

VX 10mM in propylene glycol was diluted 1:10 100µl in 876µl Phosphate Buffer 20mM pH=7.9 with 23.9µl of PCM-0212399 (6.4mg/mL, 41.8mM, in DMSO), for final concentrations of 1mM VX and 1mM PCM-0212399. Control samples were prepared similarly with 1mM VX in the same phosphate buffer with the addition of 23.9µl DMSO. At every time point reaction solution samples (triplicate) were diluted 2:998 and analyzed using the relevant MS/MS method. Measuring VX m/z 268>128, and the reaction products EMPA m/z 123>95 and isoxazole m/z 136>111. The putative pathway of

formation of the pyridine-isoxazole in the reaction of VX with PCM-0212399 is described in **Supplementary Scheme 3**.

The time-course of reaction of VX (1mM) with the amidoxime PCM-0212399 (1mM) is described in **Supplementary Figure 4**. Each MS/MS signal of the various compounds was normalized to the maximal MS/MS signal obtained in the experiment**.** The maximal MS/MS signal heights obtained are in the vicinity of 1mM VX and EMPA.

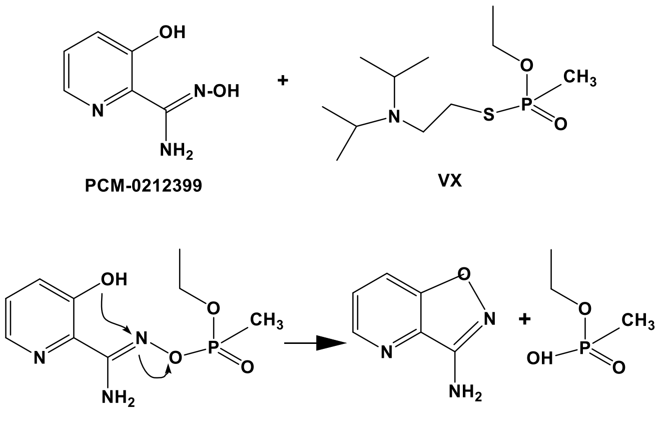

Supplementary Scheme 3: Suggested mechanism of the reaction of the amidoxime PCM-0212399 with VX first forms the corresponding phosphoryl-oxime intermediate that upon intramolecular cyclization yields pyridine-isoxzazole and O-ethyl methylphosphonic acid (EMPA). This putative mechanism is adapted from Saint-Andre et al. (2011) [3].

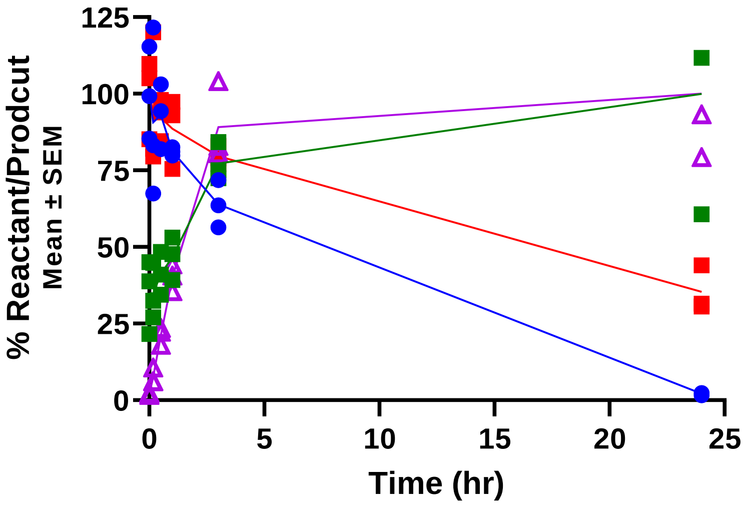

Supplementary Figure 4*:* Time-course of VX reaction with the amidoxime PCM-0212399. VX (blue circle) and the reaction products EMPA (green square) and pyridine isoxazole (empty purple triangle) were detected by LC/MS/MS analysis PCM-0212399 (red square) was reacted at 1mM with 1mM VX Relative intensity values are presented as mean ± SEM (n=3) (20mM phosphate, pH 7.9, 25°C.

4.5 Reaction of the ketoxime PCM-0211338 with Sarin analyzed by LC/MS

4.5.1 Sample preparation for LC/MS analysis

At every time point in the reaction between Sarin and the ketoxime PCM-0211338 10µl sample was diluted 10:990 phosphate buffer pH=7.0 that contains 1µL neat DMAMP in (final concentration DMAMP ~6.6mM). After 5 min reaction of Sarin with DMAMP at room temperature (derivatization reaction completion, replacing the F atom of Sarin by the phenol ester DMAMP) samples were further diluted 100:900 in phosphate buffer and stored at room temperature up to 2 hours before the LC/MS analysis

4.5.2 Establishing MS/MS transitions for PCM-0211338 and the proposed formation of phosphoryl-ketoxime intermediate

PCM-0211338 was dissolved in DMSO (0.5mg/ml, MW 270 1.85mM) then diluted in 20mM phosphate buffer (pH=7.0) to 0.2, 0.6 and 2µM. Samples were prepared at specified concentrations of Sarin and ketoxime and analyzed by an MS/MS scan with a collision energy of 24eV. It appeared that a prominent $[M+H]^+$ peak is present at Rt 7.96 minute (**Supplementary Figure 5, left**). This chromatogram peak (derived from 2µM ketoxime solution) was scanned at a collision energy of 8 and 16 eV (**Supplementary Figure 5, right**) and the MS/MS interpretation with ion fragments is presented below in **Supplementary Scheme 4**. The MS/MS transitions m/z 271->120, 271->228, 16eV were selected for the MS-MS analysis of reactants and products of the reaction of Sarin with the ketoxime PCM-0211338.

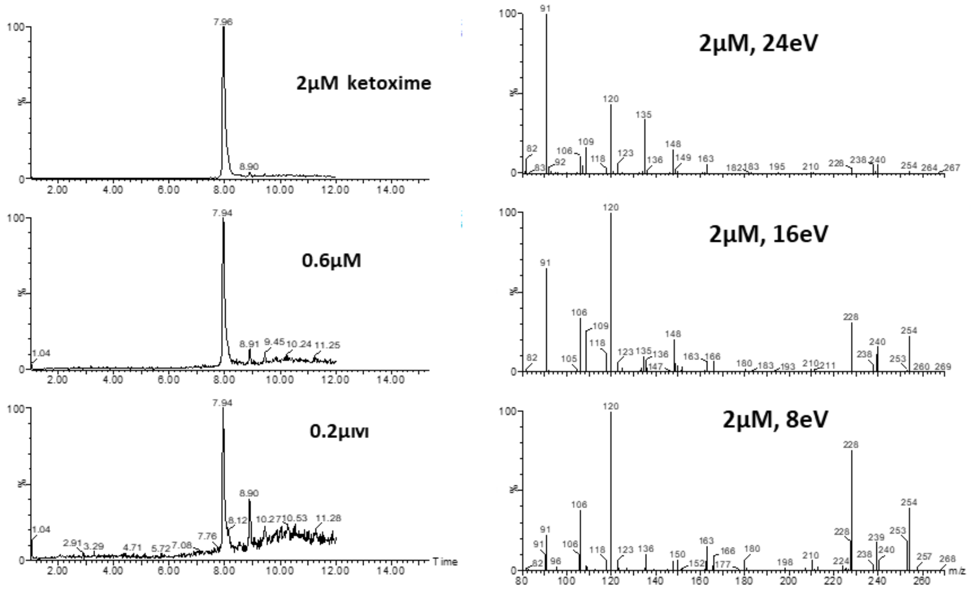

Supplementary Figure 5: LC/MS of ketoxime PCM-0211338 at 0.2, 0.6 and 2μM (left) and MS/MS scans of the 2μM PCM-0211338 LC peak at Rt 7.99 minutes at different collision energies: 8, 16 and 24eV (right)

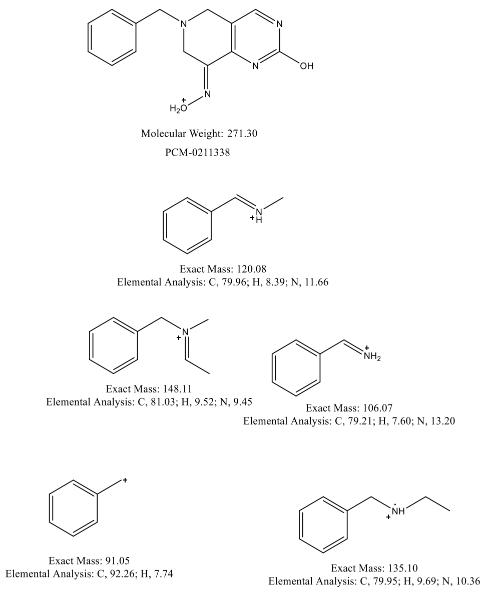

Supplementary Scheme 4*:* Proposed MS/MS fragmentation of PCM-0211338

4.5.3 Formation of Phosphoryl-Ketoxime intermediate between Sarin and the ketoxime PCM-0211338

PCM-0211338 (1mM) was reacted with 0.1, 0.3 and 1mM of Sarin in 20mM phosphate pH 7. After 30 minutes, reaction mixture samples were withdrawn and reacted with DMAMP and analyzed by MS/MS at the proposed $[M+H]^+$ Ion. It appeared that a prominent $[M+H]^+$ peak is present in the LC chromatogram at Rt 8.44 minutes (**Supplementary Figure 6 left**), which was not present in either Sarin or ketoxime control solutions (not shown). This chromatogram peak (sampled at 30 minutes of reaction of 1mM PCM-0211338 with 1mM Sarin) was scanned by MS/MS at collision energies of 8, 16 and 24 eV (**Supplementary Figure 6 right**). The proposed MS/MS assignment of the fragments is described in **Supplementary Scheme 5** and the exact mass values of the fragments were confirmed by a complementary exact mass MS/MS (Orbitrap) measurement. The MS/MS transitions m/z 391->253, 391->91, at 16eV were chosen for the MS/MS analysis of the putative phosphoryl-oxime product.

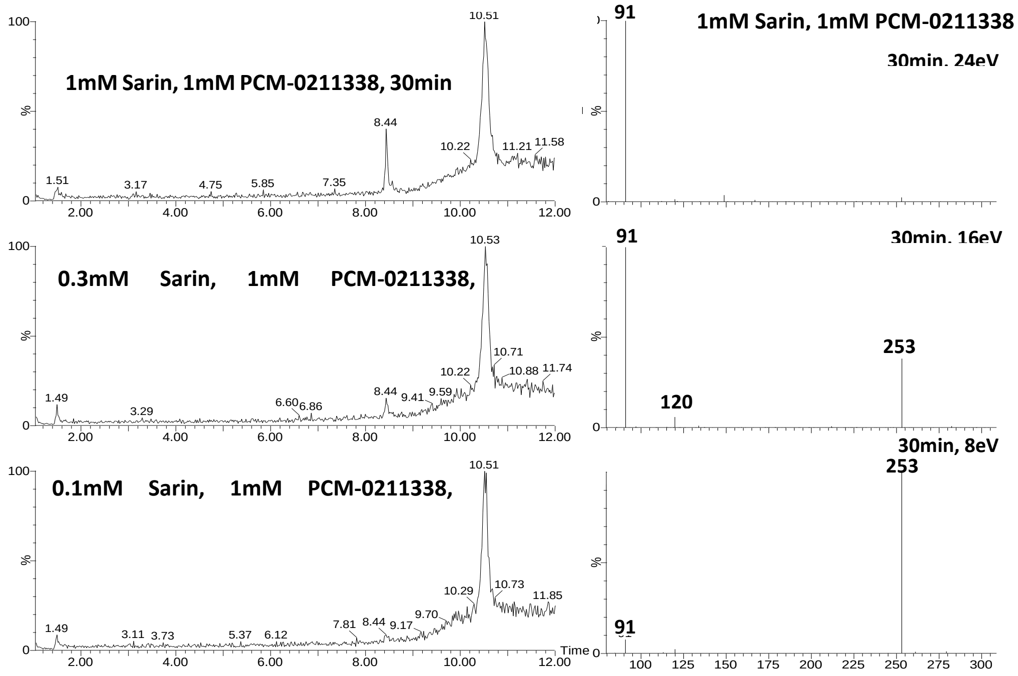

Supplementary Figure 6*:* LC/MS of the reaction product of Sarin reacting with ketoxime PCM-0211338 at 0.1, 0.3 and 1mM, sampled at 30 minutes

reaction time (left) and MS/MS scans of the LC peak at Rt 8.44 minute derived from the top left LC (1mM) at different collision energy of 8, 16 and 24eV (right)

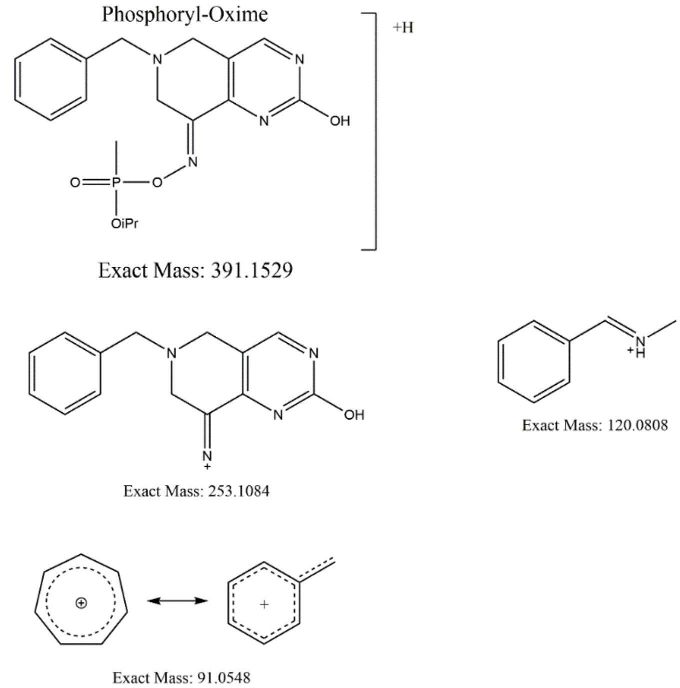

Supplementary Scheme 5*:* Proposed MS/MS fragmentation by exact mass measurement of the Phosphoryl-Ketoxime intermediate m/z 391.1529 and its Sarin

4.5.4 Time-course of reaction of PCM-0211338 (1mM) with Sarin (1mM)

PCM-0211338 (1mM) was reacted with Sarin (1mM) in 20mM phosphate buffer pH 7, 25°C. At each time point triplicate samples (10µl) were withdrawn from the reaction mixture, reacted with DMAMP for 5 minutes (as detailed above). Derivatized Sarin with DMAMP (Sarin-Der) and reaction products analyzed by MS/MS as described above. The control samples (n=3) were prepared as 1mM Sarin in 20mM phosphate buffer pH 7 with 9% DMSO (equal to the DMSO level present in PCM-0211338 1mM solution). The time-course of Sarin reaction with ketoxime PCM-0211338 is demonstrated in **Supplementary Figure 7**. Each MS/MS signal of the various compounds was normalized to the maximal MS/MS signal obtained in the experiment**.** The

maximal MS/MS signal heights obtained are in the vicinity of 1mM derivatized Sarin (Sarin-Der, **Supplementary Figure 7)** and IMPA.

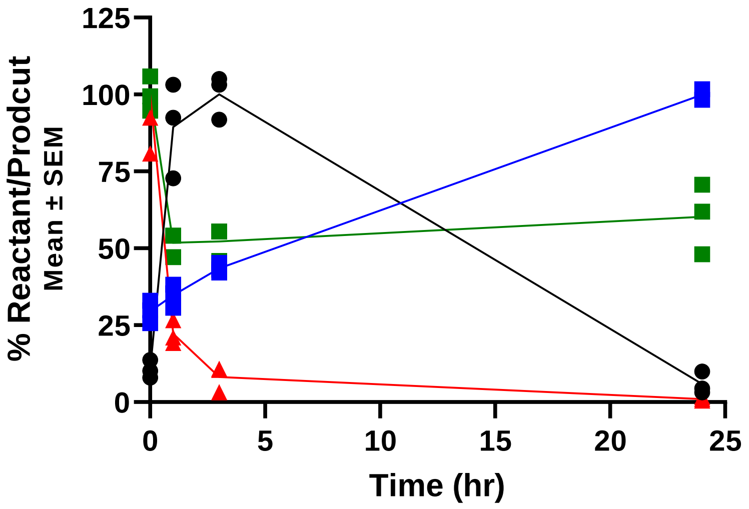

Supplementary Figure 7*:* Time-course of Sarin (1 mM) reaction with the ketoxime PCM-0211338 1 mM in 20mM phosphate pH 7.0. DMAMP Sarin-Der (red triangles) m/z 272->230 12eV PCM-0211338 (green circle) m/z 271->120 16eV Phosphoryl-Oxime (black circle) m/z 391->251 16eV. IMPA (blue square) m/z 135->95 16eV. Relative intensity values are presented as mean ± SEM (n=3). Control experiment was performed with Sarin (1mM) in 20mM phosphate pH 7, 25°C (not shown here).

Supplementary Note 5: Reactivation of Sarin- and VX-inhibited BChE by alkyne oxime analogues.

Nine alkyne analogues of HTS discovered oximes were synthesized as shown in **Supplementary Scheme 6** and then tested for their reactivation potency toward VX- and Sarin-inhibited BChE and direct detoxification of VX and Sarin.

The time-course of VX- and Sarin-BChE reactivation is depicted in **Supplementary Figure 8** and the kinetic parameters for reactivation of VX- and Sarin-inhibited BChE are summarized in **Supplementary Table 1**.

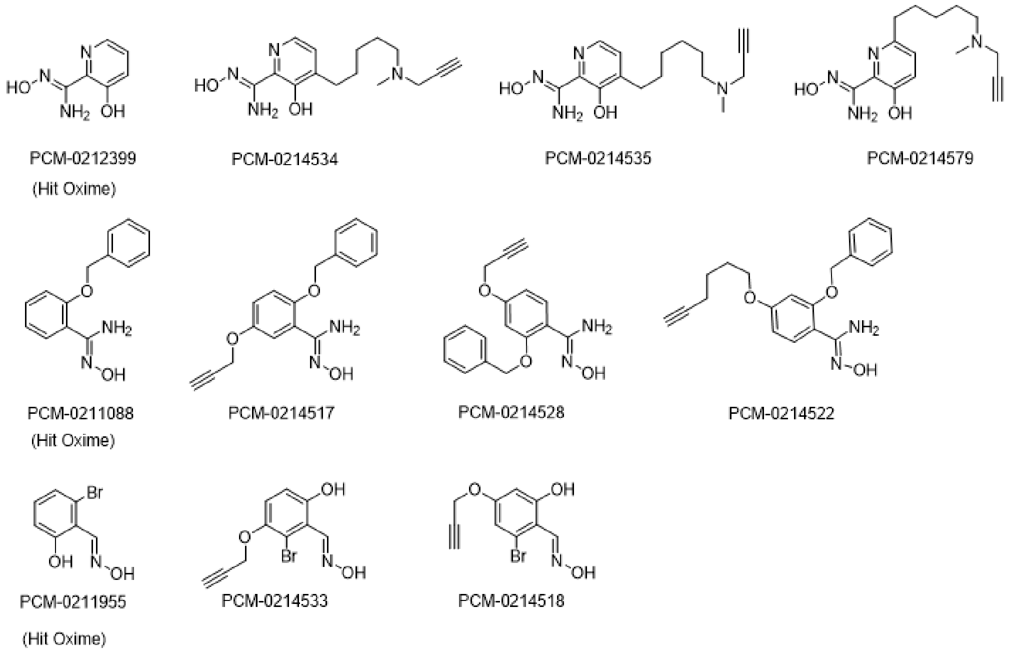

Supplementary Scheme 6*:* Chemical structure of alkyne analogues together with their corresponding hit oximes

***S*upplementary Table 1**: Kinetic parameters (k_{obs}, min^{-1}) and % maximal reactivation of VX- and Sarin-inhibited BChE by oximes and alkyne analogues (all oximes at 1mM)

Compound	VX-BChE Reactivation k_{obs}x10^{-3}, min^{-1} (% max. reactivation)	Sarin-BChE reactivation k_{obs}x10^{-3} min^{-1}, (% max. reactivation)
PCM-0211955	111 (>95%)	48 (>95%)
PCM-0214533	1.1 (90%)	0.5 (60%)
PCM-0214518	0.5 (60%)	0.2 (35%)
PCM-0212399	31 (>95%)	1.6 (90%)
PCM-0214534	34 (75%)	1.9 (45%)
PCM-0214535	4.8 (55%)	0.2 (25%)
PCM-0211088	71 (>95%)	45 (90%)
PCM-0214528	31 (65%)	21 (25%)
PCM-0214517	0.4 (50%)	18 (20%)

Notes to Supplementary Table 1: The k_{obs} values (min^{-1}) were obtained with 1mM oximes % maximal reactivation was determined at 24 hours. [VX] 1x10^{-6} M, [Sarin] 2.5x10^{-6} M, [BChE] 2.5x10^{-6}M, 20mM phosphate pH 8.0, 25°C

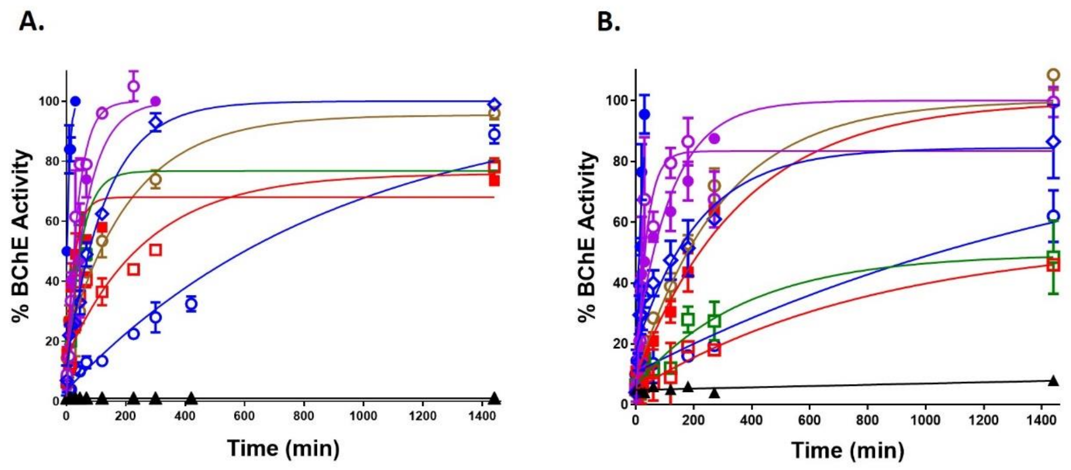

Supplementary Figure 8: Time-course of reactivation of A. VX-inhibited BChE and B. Sarin-inhibited by HTS-discovered oximes and their alkyne analogues PCM-0212399 (red filled squares), PCM-0211955 (blue filled circles) and PCM-0211088 (purple filled circles), PCM-0214528 (purple empty circles), PCM-0214535 (red empty squares), PCM-0214533 (blue empty circles), PCM-0214534 (green empty squares), PCM-0214518 (brown empty circles), PCM-0214517 (blue empty diamonds), 10mM phosphate pH 8.0 (black filled triangles), 25°C, [Oxime] = 1 mM

Supplementary Note 5: The pH-dependence of the rate of Sarin detoxification by hit oximes and one of its alkyne analogues.

Supplementary Table 2: Sarin detoxification rate constants and half-lives for HTS-selected oximes and alkyne analogue PCM-0214534 as a function of pH

**PCM**	**pH 6**		**pH 7**		**pH 8**	
	**k_{obs} (min^{-1}) x10^{3}**	**$t_{½}$ (min)**	**k_{obs} (min^{-1})x10^{3}**	**$t_{½}$ (min)**	**k_{obs} (min^{-1})x10^{3}**	**$t_{½}$ (min)**
**0212399**	15	46	137	**5**	1036	**1**
**0211955**	3	264	60	**11**	225	**3**
**0211088**	2	348	9	75	8	89
**0214534**	1	565	9	77	47	**15**
**0211338**	8	87	44	**16**	ND	-

Notes to Supplementary Table 2: Sarin (1μM) detoxification by the hit oxime PCM-0212399 and its alkyne analogue PCM-0214534 and lead oximes PCM-0211088, PCM-0211955 and PCM-0211338 [1mM] at pH 6, 7 and 8 (10mM phosphate, 25°C) (ND = not determined).

Supplementary Note 6: The pH-dependence of reactivation rate of VX- and Sarin-inhibited BChE by HTS-selected oximes and analogues. Unexpectedly, all of the HTS-identified oximes displayed the fastest rate of reactivation at pH 7 compared to pH 6 and 8 (**Supplementary Table 3**). 2PAM reactivated VX-BChE at much lower rates than the hit oximes at all pH values, as expected, and its reactivation rate was maximal at pH 8.7. In contrast to the pH-rate dependence of VX-BChE reactivation, PCM-0211955 and PCM-0212399, and the PCM-0211338 had faster reactivation rates with Sarin-inhibited BChE at pH 8 compared to pH 7 and 6 (**Supplementary Table 4**). PCM-0211088, however, had a faster rate of reactivation of Sarin-BChE at pH 7 versus pH 8.

Supplementary Table 3: The pH-dependence of the reactivation rate of VX-inhibited BChE by HTS selected oximes and alkyne analogue PCM-0214534 (initial rate constants and half-life time (k_{obs}, min^{-1}, $t_{1/2}$, min)

**PCM**	**pH 6**		**pH 7**		**pH 8/pH 8.7[(1)]**	
	**k_{obs} (min^{-1})x10^3**	**$t_{½}$ (min)**	**k_{obs} (min^{-1})x10^3**	**$t_{½}$ (min)**	**k_{obs} (min^{-1})x10^3**	**$t_{½}$ (min)**
**0212399**	1.8	378	1173	**0.6**	2.8	244
**0211955**	664	**1.0**	1651	**0.4**	1050	**0.7**
**0211088**	270	**2.6**	567	**1.2**	156	**4.4**
**0214534**	0.5	1429	0.4	1562	0.3	2570
**0211338**	0.3	1982	0.8	819	0.5	1504
**2PAM**	1.6	443	1.5	457	16	42

Notes to Supplementary Table 3: The hit oximes PCM-0211088, PCM-0211955, PCM-0211338, PCM-0212399 and its alkyne analogue PCM-0214534 were used at [1mM] at pH 6, 7 and 8. The highest rates are emphasized by red color.

(1)2PAM served as reference oxime and tested as reactivators at pH 6, 7 and 8.7 (10mM phosphate, 25°C)

Supplementary Table 4: The pH-dependence of the reactivation rate of Sarin-inhibited BChE by hit oximes and their alkyne analogues (initial rate constants and half-life time (k_{obs}, min^{-1}, $t_{1/2}$, min))

**PCM**	**pH 6**		**pH 7**		**pH 8**	
	**k_{obs} (min^{-1})x10^3**	**$t_{½}$ (min)**	**k_{obs} (min^{-1})x10^3**	**$t_{½}$ (min)**	**k_{obs} (min^{-1})x10^3**	**$t_{½}$ (min)**
**0212399**	**0.49**	**1417**	**0.75**	**920**	**1.1**	**637**
**0211955**	**8**	**89**	**19**	**37**	**31**	**22**
**0211088**	**57**	**12**	**59**	**12**	**22**	**31**
**0214534**	**0.06**	**12156**	**0.61**	**1137**	**0.47**	**1483**
**0211338**	**0.17**	**4122**	**0.24**	**2821**	**0.69**	**996**

Notes to Supplementary Table 4: The pH dependence of reactivation of Sarin-BChE was performed with hit oximes PCM-0211088, PCM-0211955, PCM-0211338, PCM-0212399 and its alkyne analogue PCM-0214534 [1mM] at pH 6, 7 and 8. The highest rates are presented by red color. (10mM phosphate, 25°C).

Supplementary References

1. Ellman, G.L., et al., *A new and rapid colorimetric determination of acetylcholinesterase activity.* Biochem Pharmacol, 1961. **7**: p. 88-95.
2. Sit, R.K., et al., *Imidazole aldoximes effective in assisting butyrylcholinesterase catalysis of organophosphate detoxification.* J Med Chem, 2014. **57**(4): p. 1378-89.
3. Saint-André, G., et al., *Design, synthesis and evaluation of new α-nucleophiles for the hydrolysis of organophosphorus nerve agents: application to the reactivation of phosphorylated acetylcholinesterase.* Tetrahedron, 2011. **67**(34): p. 6352-6361.
