# Supplementary material for: Non-quaternary oximes detoxify nerve agents and reactivate nerve agent-inhibited human butyrylcholinesterase
Source: Commun Biol. 2021 May 14;4:573. doi: 10.1038/s42003-021-02061-w (PMC8121814; doi:10.1038/s42003-021-02061-w)
Supplement: Supplementary file 2 — Description of Additional Supplementary Files [file 42003_2021_2061_MOESM2_ESM.pdf]

Description of Additional Supplementary Files

**File name:** Supplementary Data 1

**Description:** Source data for Figures 1, 2 and 3.

**File name:** Supplementary Data 2

**Description:** Source data for Supplementary Figure 3.

**File name:** Supplementary Data 3

**Description:** Source data for Supplementary Figure 4.

**File name:** Supplementary Data 4

**Description:** Source data for Supplementary Figure 7.
